# In Vitro and In Vivo Feasibility Study for a Portable VV-ECMO and ECCO_2_R System

**DOI:** 10.3390/membranes12020133

**Published:** 2022-01-22

**Authors:** Lasse J. Strudthoff, Hannah Lüken, Sebastian V. Jansen, Jan Petran, Peter C. Schlanstein, Lotte Schraven, Benjamin J. Schürmann, Niklas B. Steuer, Georg Wagner, Thomas Schmitz-Rode, Ulrich Steinseifer, Jutta Arens, Rüdger Kopp

**Affiliations:** 1 Department of Cardiovascular Engineering, Institute of Applied Medical Engineering, Medical Faculty, RWTH Aachen University, 52074 Aachen, Germany; jansen@ame.rwth-aachen.de (S.V.J.); Schlanstein@ame.rwth-aachen.de (P.C.S.); Lotte.Schraven@rwth-aachen.de (L.S.); schuermann@ame.rwth-aachen.de (B.J.S.); Steuer@ame.rwth-aachen.de (N.B.S.); Georg.wagner@rwth-aachen.de (G.W.); steinseifer@ame.rwth-aachen.de (U.S.); j.arens@utwente.nl (J.A.); 2 Department of Intensive Care Medicine, Medical Faculty, University Hospital RWTH Aachen, 52074 Aachen, Germany; hlueken@ukaachen.de (H.L.); jpetran@ukaachen.de (J.P.); rkopp@ukaachen.de (R.K.); 3 Institute of Applied Medical Engineering, Medical Faculty, RWTH Aachen University, 52074 Aachen, Germany; smiro@ame.rwth-aachen.de; 4 Department of Biomechanical Engineering, Faculty of Engineering Technologies, University of Twente, 7522 LW Enschede, The Netherlands

**Keywords:** ECMO, ECLS, ECCO_2_R, ARDS, respiratory failure, LTx, DIN EN ISO 7199, extracorporeal membrane oxygenation, acute respiratory distress syndrome, animal model

## Abstract

Extracorporeal membrane oxygenation (ECMO) is an established rescue therapy for patients with chronic respiratory failure waiting for lung transplantation (LTx). The therapy inherent immobilization may result in fatigue, consecutive deteriorated prognosis, and even lost eligibility for transplantation. We conducted a feasibility study on a novel system designed for the deployment of a portable ECMO device, enabling the physical exercise of awake patients prior to LTx. The system comprises a novel oxygenator with a directly connected blood pump, a double-lumen cannula, gas blender and supply, as well as control and energy management. In vitro experiments included tests regarding performance, efficiency, and blood damage. A reduced system was tested in vivo for feasibility using a novel large animal model. Six anesthetized pigs were first positioned in supine position, followed by a 45° angle, simulating an upright position of the patients. We monitored performance and vital parameters. All in vitro experiments showed good performance for the respective subsystems and the integrated system. The acute in vivo trials of 8 h duration confirmed the results. The novel portable ECMO-system enables adequate oxygenation and decarboxylation sufficient for, e.g., the physical exercise of designated LTx-recipients. These results are promising and suggest further preclinical studies on safety and efficacy to facilitate translation into clinical application.

## 1. Introduction

Veno-venous extracorporeal membrane oxygenation (VV-ECMO) is an established therapy for patients with severe acute respiratory distress syndrome [1]. For patients with irreversible respiratory failure, a lung transplantation (LTx) is the last resort measure [2]. For gas exchange in decompensated patients before LTx, ECMO as well as low flow extracorporeal CO_2_ removal (ECCO_2_R) has become an established strategy as a so-called bridge to (lung) transplantation [3,4,5,6]. Worldwide, 328 million patients [7] suffer from advanced chronic obstructive pulmonary disease (COPD). A growing number of patients, currently 10 million [8], suffers from advanced GOLD III/IV COPD status, resulting in 30.1% of all LTx [9,10]. Due to organ shortage, the waiting time for a suitable organ is 446 ± 517 days [11], during which time at least 10% of the patients lose therapy eligibility or decease [12,13]. Conventional treatment prior to LTx includes drug treatment and mechanical ventilation. The efficacy of this treatment is limited mostly due to ventilator induced lung injury (VILI) as well as sedation and immobilization of the patients in the intensive care unit. Immobilized patients go through a sequence of physical deteriorations comprising muscle atrophy, (poly-)neuroatrophy, and finally a state of general fatigue and deterioration of the general condition [14,15,16]. This deterioration means either losing the eligibility for or dramatically worsening the outcome of the intended LTx [17]. VV-ECMO was established as an alternative or adjuvant therapy to decrease the detrimental effects of mechanical ventilation [18]. However, the immobilization of the patients remains a problem, as current ECMO-systems are complex, heavy, bulky, require highly trained medical staff, and are prone to technical complications such as cannula kinking. The first approaches to mobilize patients have been undertaken [12,19,20,21,22,23]. The results of these studies have been promising, yet the resource intensity for the mobilization of patients is immense: Haji et al. recently published their physical exercise protocol, involving seven medical staff members for out-of-bed mobilization [24].

**Figure 1:**
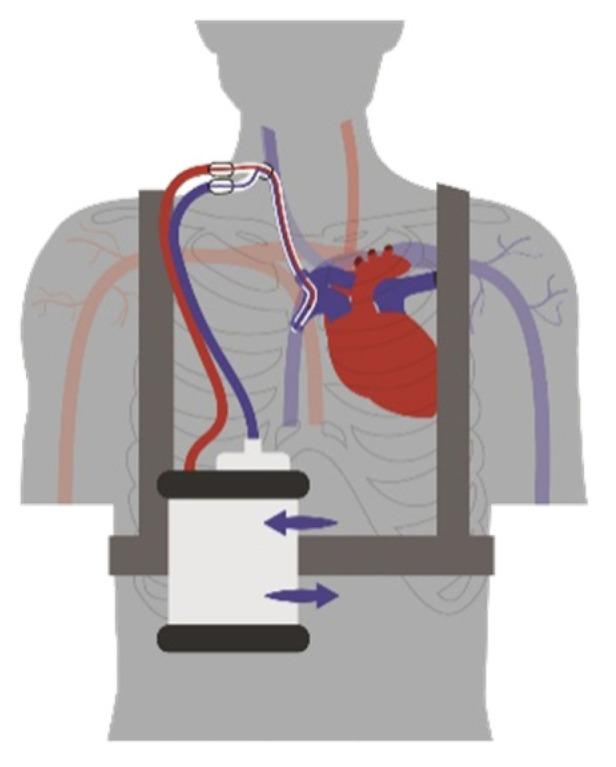
Concept for a portable VV-ECMO system.

Within the present paper, we report the results of a feasibility study for a portable VV-ECMO system. The system comprises a double-lumen cannula, an integrated pump-oxygenator-unit, portable gas supply utilizing ambient air, a battery system, and a carrying modality. The concept is depicted in Figure 1. All components were individually tested and characterized by respective in vitro tests. The entire system was tested in vivo in porcine animal trials. We demonstrated an effective system in terms of mobility, therapy-relevant parameters including gas and heat transfer, and interoperability of all components.

## 2. Materials and Methods

**Figure 2:**
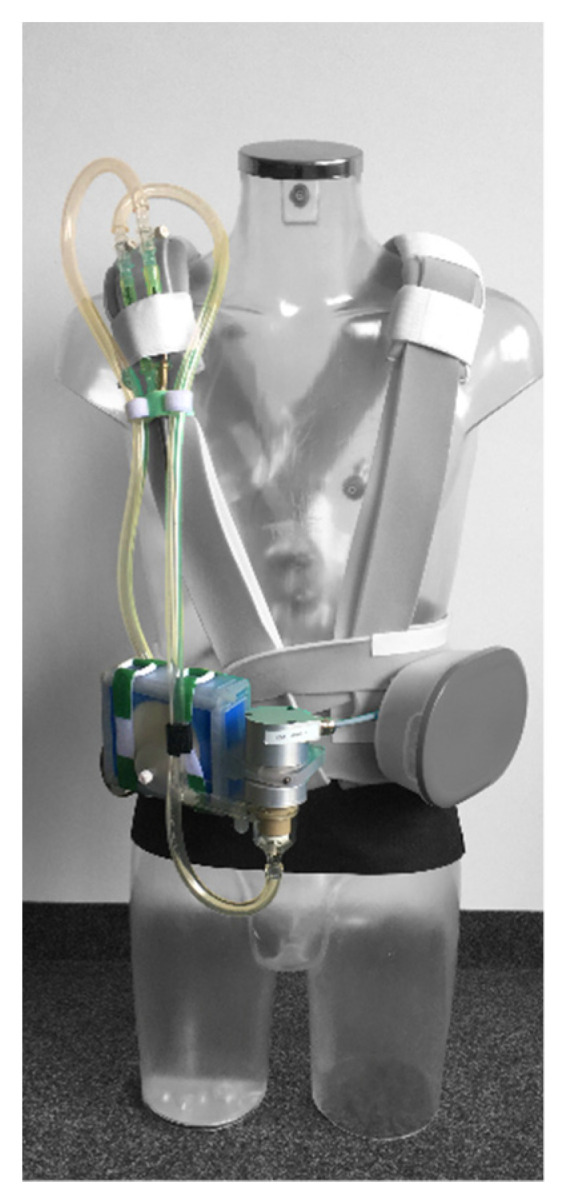
Prototype system of portable VV-ECMO system.

The portable VV-ECMO system comprises specifically designed prototypes of the oxygenator, energy supply, control unit, gas blender, and pump drive, driving a commercially available blood pump. All predefined parameters for a double lumen cannula were met by a commercially available product. All components are described below. These components were tested individually in vitro following relevant norms and guidelines (ISO 7199 [25]; Guidance for Cardiopulmonary Bypass Oxygenators 510(k) Submissions; Final Guidance for Industry and FDA Staff [26]). The integrated system was tested in a novel large animal test model. Figure 2 shows a prototype system and the intended carrying mode.

### 2.1. System Components

For the double-lumen cannula, we chose the Novaport Twin (Xenios AG, Heilbronn, Germany, now Fresenius SE & Co. KGaA, Bad Homburg, Germany). The cannula is designed for the right internal jugular access with in- and outlet situated in the superior vena cava and in front of the right atrium, respectively. The positioning of drainage and reperfusion openings of the cannula was developed to mitigate blood recirculation. With an outer diameter of 22 Fr and 3/8” connectors, the cannula was designed to allow a blood flow of up to 3 L/min with a pressure drop below 140 mmHg, based on standard requirements for VV-ECMO of the described group of patients. The cannula has a minimum kink resistance to guarantee safety during patient mobilization (<50% flow reduction during 180° kinking with kinking radius of 35 mm). Since we used a commercially available cannula, we did not evaluate the hemolysis in vitro.

As energy supply for the control system, the gas blender, and the pump drive, commercially available 11.25 V, 2.95 Ah rechargeable battery packs with three lithium-ion cells were installed (RRC2040, RRC Power Solutions GmbH, Homburg, Germany). These battery packs are characterized by quick charging, extended life span, impedance tracking, and cell balancing and do not require manual recalibration. Due to a high energy density, they have a low weight of 170 g and small dimensions of 85 mm × 59 mm × 22 mm per unit, do not produce extensive heat, and can be used in acceptable temperature ranges for patient ambulation.

As pump, a conventional DP3 with an altered pump drive was utilized (Xenios AG, Heilbronn, Germany, now Fresenius SE & Co. KGaA, Bad Homburg, Germany). The drive was miniaturized in size and weight (425 g) and, during prototype development stage, situated at the casing of the oxygenator unit. This allowed for a direct connection to the specially designed inlet of the oxygenator unit without any tubing. The DP3 pump produces a pressure head of 600 mmHg at flows far above the intended 3 L/min at max. 10,000 rpm.

**Figure 3:**
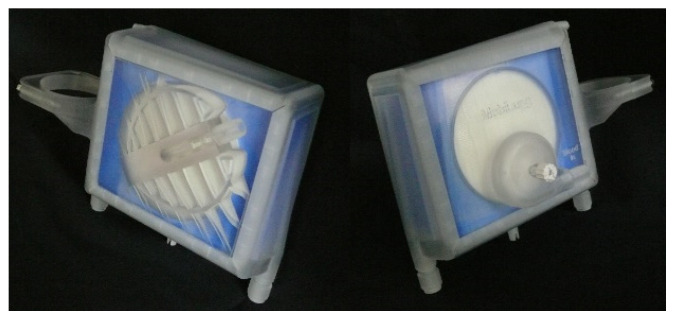
Novel portable, stacked, and centrifugal potted membrane oxygenator front and back.

The oxygenator unit was designed to achieve a flow of 3 L/min with an acceptable maximum pressure drop of around 100 mmHg (Figure 3). Further, the gas transfer efficacy should reach 150 mL/min for O_2_ and CO_2_, requiring a membrane surface of around 0.75 m^2^, based on data interpolations of existing stacked oxygenators. The fibers were arranged in stacks with solid polymethylpentene-membranes for gas transfer and polyethylenterephthalat-membranes for heat exchange (OxyPlus 90/200 single knitted loop mat and Hexpet 60/670 single distorted knitted loop mat, both Membrana GmbH, Wuppertal, Germany). Heat transfer may be necessary despite the miniaturized circuit due to extracorporeal circulation. The extent of this has not been the object of the current study. During mobilization sequences, the heat exchange fibers were not active. The phase separation was implemented using a state-of-the-art centrifugal potting process [27] with silicone (Elastosil 620, Wacker Chemie AG, Munich, Germany) and a resulting flow path diameter of 8.5 cm. The oxygenator unit also incorporates a gas bubble trap. For this early prototype, a 3D-printed casing was used. The oxygenator has a total weight of around 650 g. Targeted blood cell damage and heat transfer are within the range of commercially available VV-ECMO-systems. Corresponding experiments are described below.

**Figure 4:**
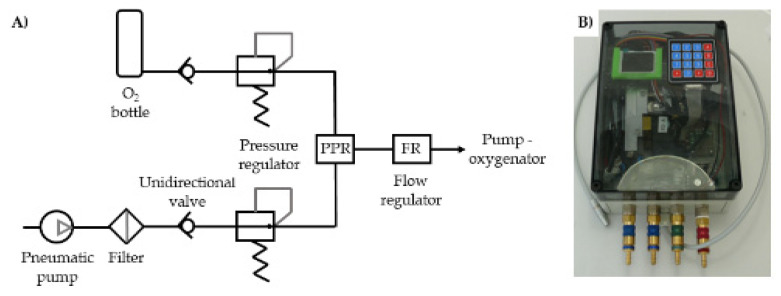
(**A**) gas blender schematic and (**B**) gas blender prototype.

The gas blender comprises pneumatic components to blend ambient air with pressurized medical oxygen including a pneumatic pump, (unidirectional) valves, air filters, pressure regulators, and a flow control system (schematic and prototype in Figure 4). The system can produce 10 L/min of flow with an F_i_O_2_ between 0% and 100% at a pressure head of 60 mmHg. The gas blender unit also contains the overall control system.

### 2.2. In Vitro Evaluation

Oxygenator pressure drops, oxygen and carbon dioxide transfer rates, heat exchanger performance factor (R-value), and blood cell damage were evaluated in vitro according to ISO 7199 [25] and FDA guideline [26]. Eleven full scale gas transfer tests were conducted with each six identical circuits including prototypes of the novel oxygenator and pooled, anticoagulated, and porcine blood. The feed gas composition and blood/gas-flow ratio were different in each of the six circuits (all possible combinations with F_i_O_2_ = 21%, F_i_O_2_ = 50%, F_i_O_2_ = 100% and 4:1 gas/blood, 1:1 gas/blood). For hemolysis testing, an iLA Membrane Ventilator in combination with a conventional DP3 pump (Xenios AG, Heilbronn, Germany, now Fresenius SE & Co. KGaA, Bad Homburg Germany) was used as predicate device in comparison to five identical prototypes of the novel oxygenator.

### 2.3. In Vivo Evaluation

To verify the function of our newly designed prototype for portable ECMO, six acute animal trials were conducted using 50–80 kg female pigs of the German Landrace. Permission for the experiments was granted by the governmental animal care committee (Landesamt für Natur, Umwelt und Verbraucherschutz Nordrhein-Westfalen, Recklinghausen, Germany, ref. no. 84-02.04.2016.A472), and the implementation followed the principles of laboratory animal care.

After arrival, the animals were examined by a veterinarian and held at least seven days in the stables of the institute of laboratory animal science of the Uniklinik RWTH Aachen for acclimatization. Twelve hours before experiment, they were kept fasting with free access to water.

The fasted pigs were premedicated with atropine (1 mL 1%), azaperone (0.2 mL/kg), and ketamine (0.1 mL/kg) and anesthetized with propofol (initial bolus 1 mg/kg and 5–10 mg/kg/h) and fentanyl (8–12 µg/kg/h). The pigs were further treated following standard protocols of intensive care. This included the treatment of electrolyte imbalances, administration of crystalloid and colloid solutions as well as medications to sustain hemodynamic stability. Throughout the trial, an ACT of ≥ 150 sec was targeted using intravenous heparin. With the onset of general anesthesia, endotracheal mechanical ventilation was initiated (tidal volume = 10 mL/kg, positive end expiratory pressure = 5 cmH_2_O, inspiratory-to-expiratory ratio = 1:2, and a respiratory frequency = 14 ± 4 min^−1^, following the target parameter P_a_CO_2_ of 35–45 mmHg). An arterial line was placed via one femoral artery, and a pulmonary arterial catheter was placed via one jugular vein to measure cardiac output, central venous pressure, and pulmonary artery pressure continuously as well as pulmonary capillary wedge pressure discontinuously (Edwards Lifesciences, Irvine, CA, USA) using an AS/3 Compact monitor (Datex-Ohmeda, Helsinki, Finland) and a Vigilance monitor (Edwards Lifesciences). A bladder catheter was maintained to collect urine for the control of the renal function. The animals received infusions of 1 mL/kg/h Sterofundin (B.Braun, Melsungen, Germany) for fluid replacement. As pigs have a much larger diameter of the external jugular vein compared to the internal jugular vein, the right external jugular vein was cannulated with the NovaPort dual lumen cannula. After one bolus of 5000 IU Heparin, the prefilled ECMO was connected and blood flow was started.

**Figure 5:**
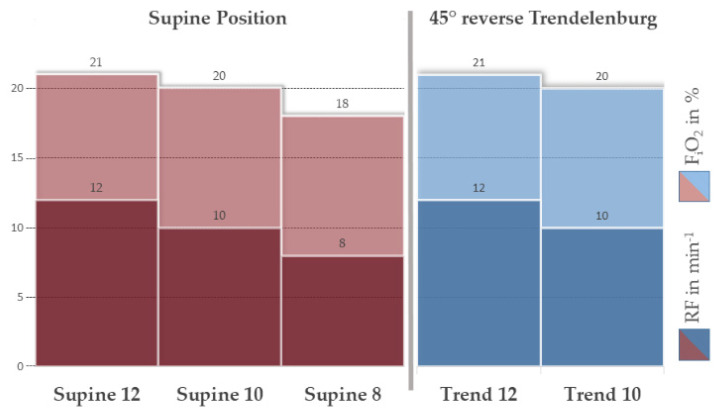
Experimental sequence plan. Fully anesthetized pigs were supported by intermittent mechanical ventilation with an initial respiratory rate >12 min^−1^ and F_i_O_2_ of >21%. The sequence plan shows incremental decreases of these values to model hypercapnic and hypoxic respiratory failure. After holding each plateau for 30 min, blood samples were taken. ECMO-support was adjusted to stabilize the vital parameters for each plateau. During the first three measurement points, the animal was lying in supine position, followed by a simple simulation of a mobilized patient by changing to 45° reverse Trendelenburg position (see Figure 6). These groups are referred to throughout the manuscript: Supine: animal was positioned flat on the back; Trend: 45° reverse Trendelenburg position, simulating a mobilized patient; and 12,10,8: respiratory rate of the intermittent mechanical ventilation, simulating hypercapnic respiratory failure.

The experimental sequence plan is depicted in Figure 5 and described in the following paragraph.

The experimental protocol provided an 8 h timeline from ECMO connection with fixed time points of measurements, blood samplings, and mode changing from lying to upright position and back. The animal model contains an induced hypercapnic and hypoxic respiratory failure by lowering the F_i_O_2_ and the respiration frequency using the P_a_O_2_ and P_a_CO_2_ as target parameters, originally published by Kopp et al. 2016 [28]. First, animals were ventilated with an F_i_O_2_ of 0.21 and a respiratory rate of 12 min^−1^. By reducing the F_i_O_2_ to 20% and 18% and, correspondingly, the respiratory rate to 10 and 8 min^−1^, we generated a standardized hypoxemia and hypercapnia to simulate respiratory failure. The ECMO was running as low as possible and was adjusted according to the respective measured values with a target arterial oxygen saturation of ≥ 90% and a target arterial carbon dioxide partial pressure of 40–50 mmHg. Blood gas analysis and hemodynamic parameters were measured every 30 min, before further reduction of F_i_O_2_ and respiratory rate. During this process, the ECMO was initially tested running with ambient air; then, the feed gas was blended with oxygen to meet the physiologic oxygen demand.

**Figure 6:**
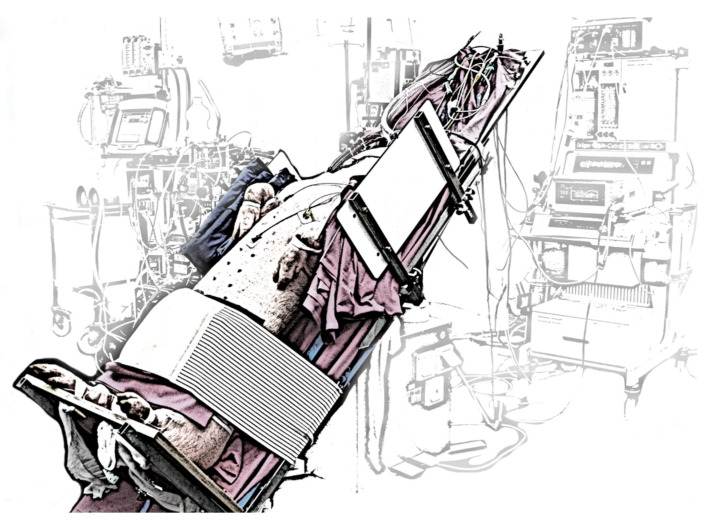
45° Reverse Trendelenburg position with abdominal compression. The animals were placed on the operating table, a robust board was mounted below their back hooves, and the compression strap was tightened around the animal abdomen and the operating table.

The test protocol contained two distinctions: First, the pigs were brought up from supine position to a 45° reverse Trendelenburg position, simulating an upright patient as well as possible, taking into account the limitations of acute large animal models (represented on right side of Figure 5, depicted in Figure 6). In this model, vasoplegia and hence dilated veins during general anesthesia in combination with gravity may cause a distributive shock. This problem was counteracted by decreasing the passive volume of the abdominal venous reservoir by abdominal compression. Vasopressors were discarded as an option as their effect is mostly on arterioles and not the venous system which impacts the blood pressure but does not solve the targeted venous pooling. The second distinction regards the blending of medical oxygen with ambient air. The feed gas was altered during the course of the experiments, to see the impact of lower F_i_O_2_. Upon experiment finalization, animals were euthanized.

During the in vivo experiments, the system and its components were tested on overall handling and performance, including vital parameters of the pigs. Oxygenator and pump unit were tested regarding gas transfer, pressure heads and drops, and hemolysis. Gas transfer was tested taking blood samples pre- and post-oxygenator. Hemolysis was tested every half-hour to calculate the normalized index of hemolysis (NIH) without a predicate reference device.

### 2.4. Statistical Analysis

For in vitro experiments, all results are depicted with mean and standard deviation (SD). Hemolysis values from the in vitro experiments were tested for significance comparing the five test objects against the single control circuit. First, normal Gaussian distributions were tested using the Kolmogorov and Smirnov method. *p*-values above 0.10 were considered normal Gaussian distributions. Then, we conducted a two-tailed one sample *t*-test; a *p*-value < 0.05 was considered significant.

The in vivo experiments were evaluated by calculating median, mean, and standard deviation. If more than one sample point was recorded within a group, their mean was calculated before the actual evaluation. All parameters shown in the result section (Table 1) were tested for significant differences between the five groups of the experimental sequence plan (Figure 5) using the Friedmann test with post Dunn’s test. Two animals had no data in a single group; these values were imputed using the mean of the remaining group. If more than one value was missing for a single parameter, an unpaired Kruskal–Wallis test was conducted instead. Here, a post-test was not required due to insignificant results. For all tests, a *p*-value < 0.05 was considered significant.

## 3. Results

The resulting system is depicted in Figure 2. All predefined criteria for the portable utilization for ECMO-patients were met. The system showed efficacy during in vitro experiments and in vivo trials.

### 3.1. In Vitro Evaluation

**Figure 7:**
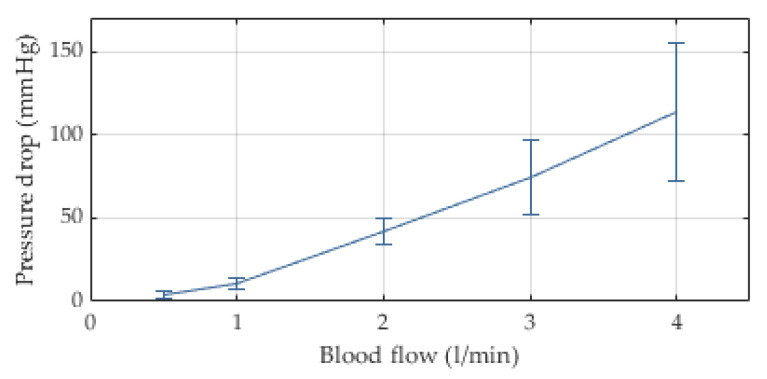
In vitro pressure drop in novel stacked membrane oxygenator using heparinized porcine blood, n = 5, mean and SD.

**Figure 8:**
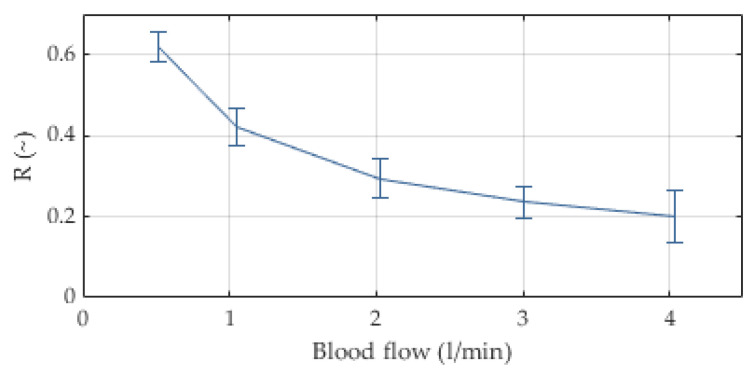
Heat transfer efficacy depicted as R-value (following the test protocol in ISO 7199), n = 10, mean and SD.

**Figure 9:**
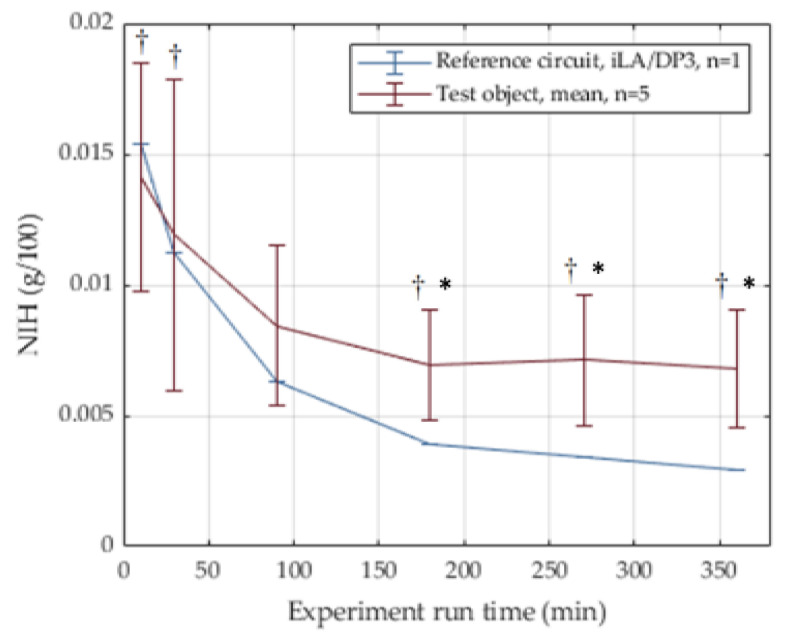
In vitro hemolysis results depicted as normalized index of hemolysis (NIH), mean and SD. The predicate device is depicted in blue (n = 1, iLA oxygenator, DP3 pump), and the test devices are depicted in red (n = 5). †: Normal distribution (tested with Kolmogorov–Smirnov), *: significant difference between test device and predicate (two-tailed one sample *t*-test, *p* < 0.05 for Gaussian distributions). The non-Gaussian distribution at t = 90 min was not further analyzed due to small sample size.

The internal resistance of the extracorporeal circuit to blood flow is mainly caused by the cannula (properties available from manufacturer) and the oxygenator. The stacked fiber mat design results in a pressure drop of around 75 mmHg (±20) at a blood flow rate of 3 L/min, see Figure 7. The integrated heat exchanger fibers allow for a heat transfer of R = 0.24 (±0.04) at the same blood flow; see Figure 8. The in vitro blood tests yielded a threefold normalized index of hemolysis (NIH) of the novel device compared to a reference device (Figure 9).

**Figure 10:**
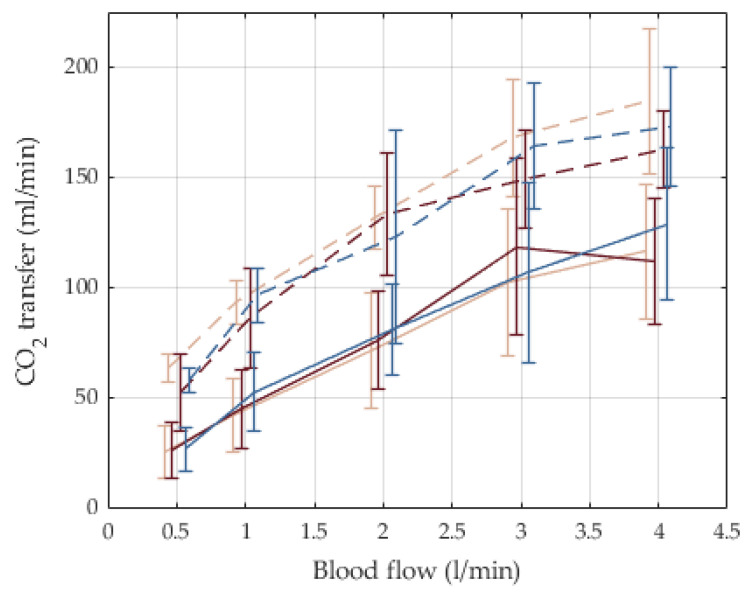
Carbon dioxide transfer rates for different feed gas blends and gas/blood-flow ratios, n = 5, mean and SD. Beige: feed gas with pure ambient air, F_i_O_2_ = 21%; red: feed gas blend with F_i_O_2_ = 50%; blue: feed gas with medical oxygen, F_i_O_2_ = 100%. Solid line: 1:1 flow ratio gas/blood; dashed line: 4:1 flow ratio gas/blood.

**Figure 11:**
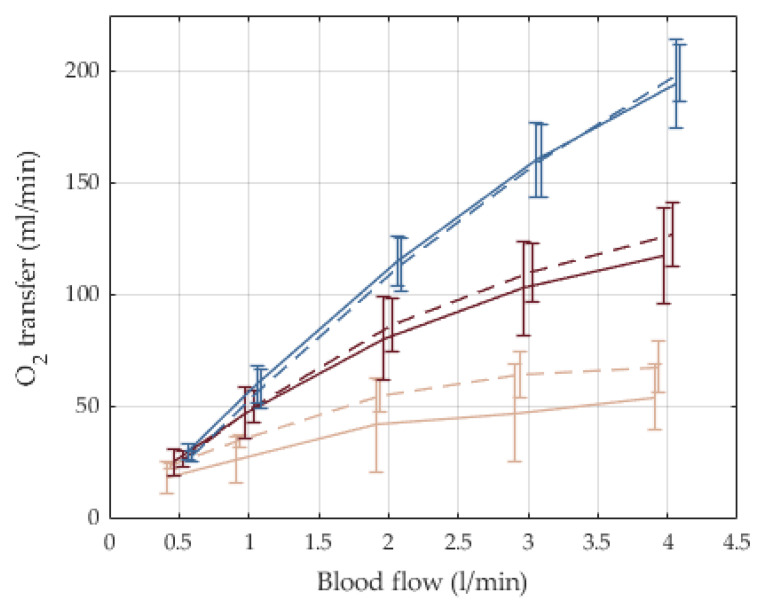
Oxygen transfer rates for different feed gas blends and gas/blood-flow ratios, n = 5, mean and SD. Beige: feed gas with pure ambient air, F_i_O_2_ = 21%; red: feed gas blend with F_i_O_2_ = 50%; blue: feed gas with medical oxygen, F_i_O_2_ = 100%. Solid line: 1:1 flow ratio gas/blood; dashed line: 4:1 flow ratio gas/blood.

Carbon dioxide and oxygen transfer rates are depicted in Figure 10 and Figure 11, respectively. CO_2_ transfer rates at 3 L/min were 110 mL/min with a flow ratio for gas/blood flows = 1:1. The transfer rates increase to 160 mL/min with a ratio for gas/blood flow = 4:1. The rates were almost independent of the oxygen fraction. The oxygen was transferred with 50, 110, and 160 mL/min for oxygen fractions of 21%, 50%, and 100%, respectively. The oxygen transfer was almost independent of the ratio for gas/blood flows.

### 3.2. In Vivo Evaluation

A total of six pigs were put on the dedicated ECMO system and treated following the protocol described above. All animals survived until the planned termination of the experiment. Five animals had stable vital parameters during each stage, reaching a respiratory rate as low as 8 min^−1^ and a mean F_i_O_2_ of 18%. The sixth animal did not reach a respiratory rate of 8 min^−1^ in the supine position but in the Trendelenburg position. Throughout all experiments, the heart rate remained stable for three of the five experimental stages and increased during the last sequences of both supine and 45° reverse Trendelenburg position. The mean arterial pressure showed no conclusive trend. The mean pulmonary artery pressure changed only due to the 45° reverse Trendelenburg positioning, by an average decrease of 10 mmHg. Due to anesthesia and the resulting lack of vasoconstriction, sufficient vital parameters could not be sustained below a respiratory rate of 10 min^−1^ during the 45° reverse Trendelenburg position, despite abdominal compression. The ACT was kept above 150 s as targeted. Neither bleeding nor macroscopically or clinically observable thrombosis occurred during the experiments. The hemoglobin was mostly kept between 9 and 11 g/dL. There was no technical failure with any of the components.

Table 1 shows the measured data from intermittent mechanical ventilation, vital parameters, ECMO settings, and blood gas analysis.

**Table 1:** Animal trial monitoring. Supine: animal was positioned flat on the back; Trend: 45° reverse Trendelenburg position, simulating a mobilized patient; and 12,10,8: respiratory rate of the intermittent mechanical ventilation, simulating hypercapnic respiratory failure. RMV = respiratory minute volume; F_i_O_2_ = fraction of inspired oxygen, utilized for simulating hypoxemic respiratory failure; HF = heart frequency; MAP = mean arterial pressure (approximated from end-systolic and end-diastolic pressures); MPAP = mean pulmonary arterial pressure (approximated); S = oxygen saturation, p = partial pressures, et = end tidal/respiratory, ΔP = membrane pressure drop; fPHb = free plasma hemoglobin; and V = gas transfer. Indices: a = arterial, v = mixed venous at pulmonary artery, and cv = venous at vena cava superior. Carbon dioxide blood contents were calculated using a function by Douglas et al. [29] with a CO_2_-plasma diffusion coefficient of 0.03 mmol/mmHg and an apparent dissociation constant of carbonic acid of 6.1. Oxygen contents were calculated using a function by Leach et al. [30] with the Hüfner-number = 1.34 and with an O_2_-plasma diffusion coefficient of 0.03 mL/mmHg/dL. Significance was tested for all groups using the Friedman test ^(^*^)^ with Dunn’s post-test. ^(0)^: No significance between any combination of groups using Dunn’s; ^(1)^: Supine 8 was significantly different to Supine 12 and Trend 12 using Dunn’s. If values were missing within a parameter set, Kruskal–Wallis test was conducted ^(†)^. No parameter and combination of tested groups showed significance for *p* < 0.05, except for the controlled parameter F_i_O_2_. The column *p*-value shows the Friedman/Kruskal–Wallis results.

		Supine 12			Supine 10			Supine 8			Trend 12			Trend 10			*p*-Value
Parameter	Unit	Mean	Median	SD	Mean	Median	SD	Mean	Median	SD	Mean	Median	SD	Mean	Median	SD	
RMV	L/min	4	5	2	4	4	1	3	3	2	5	5	1	5	4	1	0.040 *^, (0)^
F_i_O_2_	%	21	21	1	20	20	3	18	18	0	21	21	1	20	19	1	0.004 *^, (1)^
HF	1/min	119	111	34	119	121	51	151	169	56	166	168	40	171	171	36	0.035 *^, (0)^
MAP	mmHg	75	71	14	105	94	38	94	84	18	79	75	20	71	71	15	0.040 *^, (0)^
MPAP	mmHg	22	20	8	24	21	10	24	23	5	14	14	12	17	15	16	0.355 *^, (0)^
S_a_O_2_	%	84	87	11	80	85	14	73	77	24	83	87	10	73	74	11	0.483 ^†^
S_cv_O_2_	%	71	72	10	61	67	13	52	61	17	56	56	7	57	54	11	0.235 ^†^
S_v_O_2_	%	65	67	6	68	72	13	55	62	18	57	58	5	56	55	4	0.231 ^†^
p_a_O_2_	mmHg	66	64	15	67	67	19	54	50	19	62	65	10	52	50	8	0.311 ^†^
p_cv_O_2_	mmHg	49	47	6	43	43	5	60	40	35	40	42	3	41	40	4	0.157 ^†^
p_v_O_2_	mmHg	44	44	2	50	45	12	38	39	5	40	41	3	40	40	3	0.075 ^†^
p_a_CO_2_	mmHg	53	55	5	49	50	8	49	45	13	50	49	7	53	51	8	0.556 ^†^
p_cv_CO2	mmHg	58	58	5	57	58	6	56	48	16	57	56	6	59	55	10	0.873 ^†^
p_v_CO_2_	mmHg	56	55	5	52	51	7	51	47	16	54	53	7	57	56	9	0.744 ^†^
etCO_2_	%	7	6	1	6	7	2	7	6	1	7	7	1	7	7	1	0.747 *
Arterial lactate	mmol/L	2.8	1.0	4.1	3.5	1.7	4.1	3.0	1.8	2.7	4.1	2.6	4.0	5.6	4.5	4.8	0.255
Arterial pH	[]	7.4	7.4	0.1	7.4	7.4	0.1	7.4	7.4	0.1	7.4	7.4	0.1	7.3	7.3	0.1	0.321 ^†^
Blood flow ECMO	L/min	1.3	1.7	0.7	1.8	1.9	0.7	2.3	2.2	0.7	1.4	1.6	0.8	2.0	1.9	0.4	0.045 *^, (0)^
Pump speed	min^−1^	5470	5617	2878	6803	7188	2287	7457	7992	1774	5797	6967	2647	8097	8100	1581	0.082 *
Gas flow	L/min	4	4	4	5	4	3	6	6	3	4	4	3	5	5	3	0.749 *
F_i_O_2_ ECMO	%	68	100	43	86	100	23	100	100	0	74	100	41	100	100	0	0.171 *
ΔP ECMO	mmHg	7	7	13	18	20	14	30	28	16	12	16	15	23	26	10	0.075 *
fPHb	mg/dL	15	13	6	12	12	7	12	12	7	14	12	4	13	14	6	0.920 *
SO_2_ post ECMO	%	96	100	8	100	100	1	98	100	5	98	100	3	100	100	0	0.922 *
SO_2_ pre ECMO	%	69	76	10	63	68	12	55	58	14	64	68	9	56	55	5	0.323 *
pO_2_ post ECMO	mmHg	278	322	199	239	227	86	223	182	125	228	232	139	234	181	107	0.736 *
pO_2_ pre ECMO	mmHg	48	50	5	48	45	11	52	40	27	45	46	4	41	40	2	0.171 *
pCO_2_ post ECMO	mmHg	38	37	13	36	36	5	40	36	7	37	36	3	41	41	3	0.073 *
pCO_2_ pre ECMO	mmHg	58	60	5	54	56	8	54	51	11	55	55	6	59	57	8	0.294 *
VCO_2_ ECMO	mL/min	106	69	126	85	86	28	78	88	41	58	57	29	60	55	20	0.255 *
VO_2_ ECMO	mL/min	56	57	48	90	93	49	122	133	45	69	74	46	116	123	32	0.220 *

**Figure 12:**
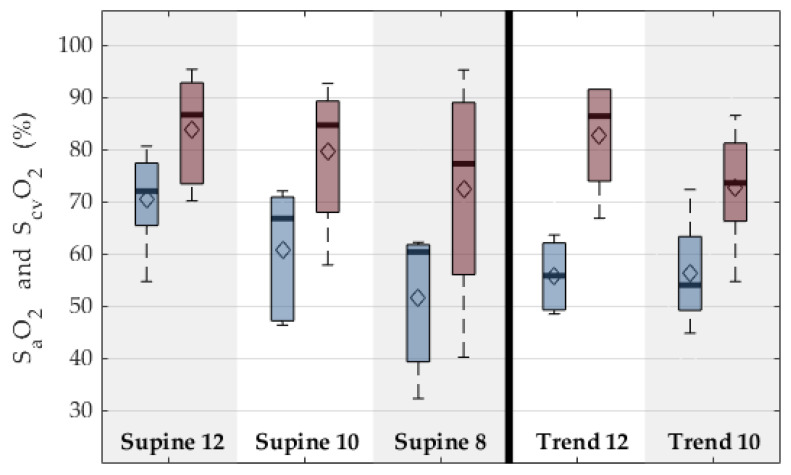
Mixed venous and arterial oxygen saturations for supine position and reverse Trendelenburg. Each column depicts one group of the experimental sequence. Supine: animal was positioned flat on the back; Trend: 45° reverse Trendelenburg position, simulating a mobilized patient; and 12,10,8: respiratory rate of the intermittent mechanical ventilation, simulating hypercapnic respiratory failure. Blue depicts venous values, and red depicts arterial values. Boxplots show 95%-CI (whiskers), 50% interval (box), median (black bar), and mean (diamond).

The ECMO blood flow was mostly well below its functional limit, with a maximum flow of 3 L/min for a total of only 2 h in a single animal. A higher blood flow could not be achieved during the experiments due to a combination of drainage insufficiency and suction at the small double lumen cannula. This resulted in very low arterial oxygen saturation S_a_O_2_ between 40% and 95%. It can also be seen that the venous saturations S_v_O_2_ are very low as well and decreasing with progression through the experimental sequences. Figure 12 shows the difference between arterial and venous oxygen saturation in all experimental sequences. The course of the oxygen saturation and the lactate levels suggest a progressing oxygen debt. At the same time, the blood gas analysis at the oxygenator outlet showed saturations well above 90% while partial oxygen pressures at the inlet mostly were between 40 and 70 mmHg. The oxygen transfer over the membrane oxygenator stays above 56 mL/min on average and increases to an average of 122 mL/min for higher ECMO blood flows. The respective carbon dioxide elimination ranges between 58 and 106 mL/min.

**Figure 13:**
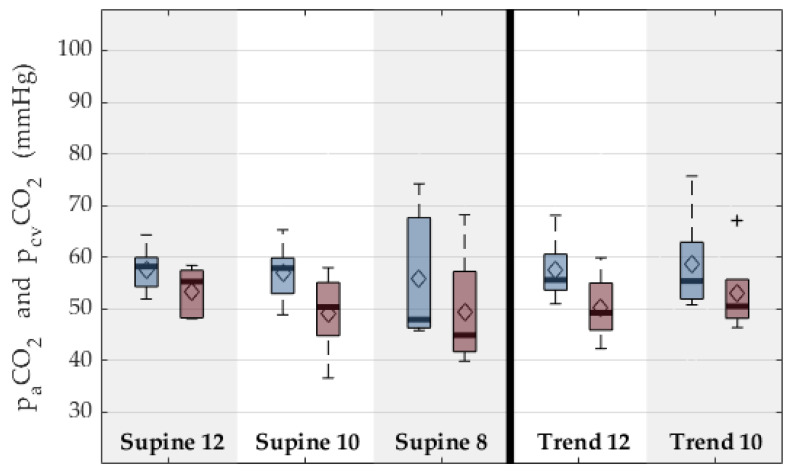
Venous and arterial carbon dioxide partial pressures for supine position and reverse Trendelenburg. Each column depicts one group of the experimental sequence. Supine: animal was positioned flat on the back; Trend: 45° reverse Trendelenburg position, simulating a mobilized patient; and 12,10,8: respiratory rate of the intermittent mechanical ventilation, simulating hypercapnic respiratory failure. Blue depicts venous values in the vena cava, and red depicts arterial values. Boxplots show 95%-CI (whiskers), 50%-interval (box), median (black bar), mean (diamond), and outlier (black cross).

In contrast to the O_2_-values that decrease with progression of the experimental sequences, the venous p_cv_CO_2_-levels stay relatively stable between 45 and 75 mmHg. The CO_2_-elimination even increases with progression of the experimental sequences in supine position and decreases in 45° reverse Trendelenburg position. Figure 13 shows the courses of the venous and arterial p_cv_CO_2_. Simultaneously, the pCO_2_-elimination in the ECMO-circuit effectively lowers the pCO_2_ from between 50 and 65 mmHg in the drainage lumen of the cannula to around 25–45 mmHg in the return lumen. Throughout all experiments, the pH-values mostly remained between 7.2 and 7.5.

**Figure 14:**
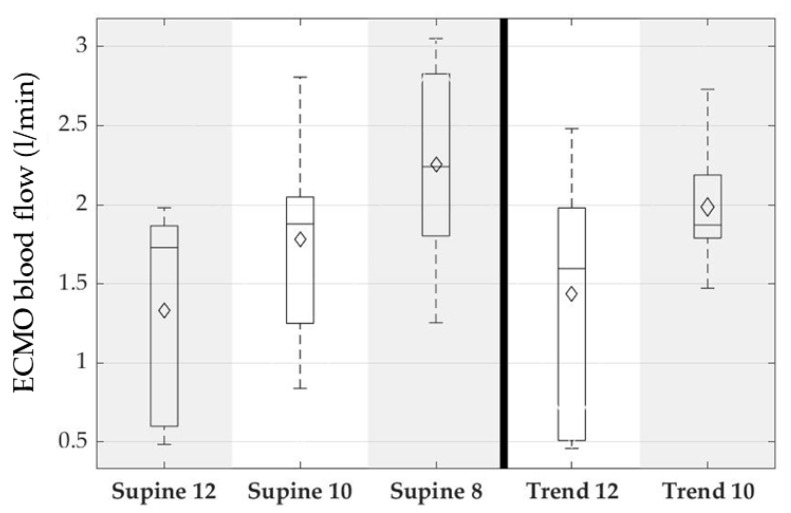
ECMO blood flow for supine position and reverse Trendelenburg. Each column depicts one group of the experimental sequence. Supine: animal was positioned flat on the back; Trend: 45° reverse Trendelenburg position, simulating a mobilized patient; and 12,10,8: respiratory rate of the intermittent mechanical ventilation, simulating hypercapnic respiratory failure. Boxplots show 95%-CI (whiskers), 50%-interval (box), median (black bar), and mean (diamond).

**Figure 15:**
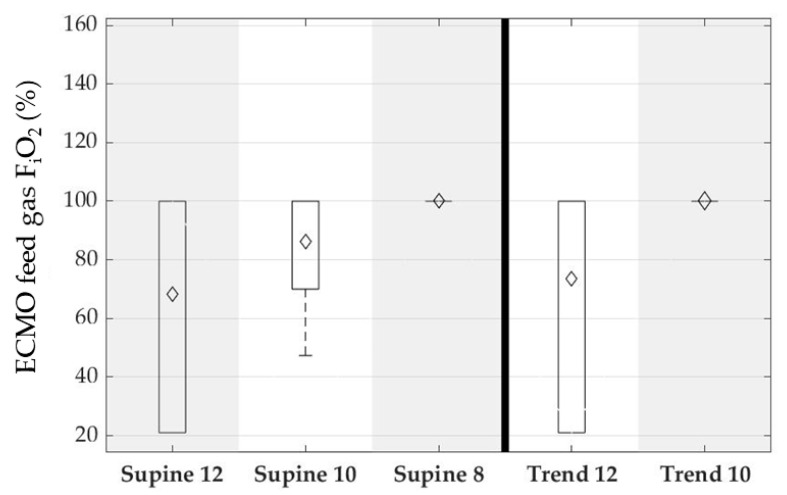
ECMO feed gas oxygen fraction for supine position and reverse Trendelenburg. Each column depicts one group of the experimental sequence. Supine: animal was positioned flat on the back; Trend: 45° reverse Trendelenburg position, simulating a mobilized patient; and 12,10,8: respiratory rate of the intermittent mechanical ventilation, simulating hypercapnic respiratory failure. Boxplots show 95%-CI (whiskers), 50%-interval (box), median (black bar), and mean (diamond).

The ECMO parameters reflect the cannula related issues to achieve a flow of 3 L/min and the concept to run the ECMO circulation as low as possible. The ECMO blood flow was increased with the reduction of ventilator settings from on average 1.3 L/min to 2.3 L/min and ranged between 500 mL/min and 3 L/min. Figure 14 shows the corresponding boxplots. Moreover, it was intended to run the ECMO with initial oxygen fractions of ambient air and increase with physiologic demand. It can be seen that the oxygen fraction was increased early to 100% with decreasing ventilator settings. Figure 15 shows the ECMO oxygen fraction over the experimental sequence as boxplots.

Finally, the pressure drop in all six oxygenators proved to be around 60% of the values determined in vitro, which means that the pressure drop at 3 L/min was between 40 and 50 mmHg drop (compared to 50–100 mmHg in vitro). Hemolysis was measured as change of free plasma hemoglobin and showed no increase in any animal. Values stayed in a range of 4–22 mg/dL.

## 4. Discussion

A novel veno-venous ECMO system with downsized and integrated components dedicated for patient mobilization was tested in vitro and in vivo.

### 4.1. In Vitro Evaluation

The system was tested for hemolysis and membrane pressure drop, as well as heat and gas transfer for the intended use case. The use case of the device was defined as VV-ECMO for a blood flow of up to 3 L/min and ECCO_2_R. Comparable, commercially available devices for VV-ECMO are the HLS Set Advanced 5.0 (Maquet Cardiopulmonary GmbH, Rastatt, Germany) while one suitable device for ECCO_2_R may be the novalung iLA (Xenios AG, Heilbronn, Germany). Both oxygenators have a stacked fiber arrangement. At 3 L/min and a flow ratio gas/blood of 1:1, the HLS oxygenator has an O_2_-transfer rate of almost 200 mL/min, a CO_2_-transfer rate of approximately 170 mmHg, a heat-exchange performance factor of approximately 0.8, and a pressure drop of 10 mmHg [31]. The iLA does not contain heat fiber mats. The O_2_-transfer rate is approximately 160 mL/min, the CO_2_-transfer is approximately 140 mL/min, and the pressure drop is 10 mmHg as well [32]. We do not have published data on hemolysis performance for both devices but used the iLA oxygenator in combination with a DP3-pump as a predicate device during in vitro experiments.

Heat and gas transfer achieved targeted values, though approximately at 25% of the efficacy of the HLS 5.0. This may be acceptable as the extracorporeal circuit has a lower blood volume, blood flow, and less unintended surface area, resulting in smaller heat loss; the same concept can be found in the iLA oxygenator that has no heat exchange fibers at all. Target oxygen transfer rates were realized only at pure oxygen for both 1:1 and 4:1 flow ratio gas/blood. Target CO_2_-transfer rates were reached only with 4:1 flow ratio, independent of oxygen fraction. These results were anticipated, though lower required oxygen fractions would strengthen the use case of the device. All gas transfer rates are comparable to the iLA oxygenator, which means approximately 75% of the HLS 5.0. Furthermore, Ficial et al. [33] recently hypothesized that a higher F_i_O_2_ in the ECMO circuit may translate into higher CO_2_ transfer rates by displacing CO_2_ from HCO_3_^−^; our data do not support this theory, at least not in clinically relevant dimensions.

The pressure drop with respect to commercial devices was approximately 800% higher in vitro and 300% in vivo; the reason of which remains unclear but could be explained with the laboratory grade prototype status of the novel oxygenator and its evolution over the progression of the research project. We hypothesized based on institutional experience that fiber arrangement and density are very sensitive to flow resistance—a problem of less extent in industrial grade production. Hemolysis was three times as high as in the predicate device. Again, the prototype status of the oxygenator may have caused a significant increase in hemolysis, because the entire casing was printed. Recently, Petersdorff-Campen et al. [34] showed an increase in hemolysis in printed blood pumps of 620%, which may explain our results. Further, the higher resistance to flow and the hence increased pump performance may have caused hemolysis. Future improvements to the integrated system of pump and oxygenator will target the increased pressure drop and hemolysis. We see no technology inherent disqualifying criterion to designing a safe system. Advances to the manufacturing process, material selection, flow path optimizations of pump, oxygenator and connecting parts, and a pump optimized to the targeted pump performance regime will reach safe pressure drop and hemolysis levels.

Last, we have successfully used a portable gas blender that allows quick, individual, and automatable gas mixtures of medical oxygen and ambient air.

### 4.2. In Vivo Evaluation

The in vivo model was designed with three characteristic features. First, the hypoxic and hypercapnic respiratory failure was simulated using the respiratory rate and oxygen fraction of the intermittent mechanical ventilator. Both hypoxia and the hypercapnia could effectively be established in a controllable and stable manner. Second, the animal was switched from supine position to 45° reverse Trendelenburg position, with the aim to simulate an upright awake patient. Established animal models for ECMO testing use the recumbent position [35] or sometimes quadruped position [36,37], which do not reflect the hemodynamic changes of an upright patient. The anesthesia prohibited effective physiologic autoregulation, especially vasoconstriction, so that we actually simulated a distributive hypovolemic shock. We had to counteract this shock via the experiment design. Vasopressors were discarded as their effect mainly targets the arterioles and does not resolve venous pooling. Instead, we applied an abdominal cingulum compression. This model is not yet established through these experiments, but we showed efficacy and feasibility. Additionally, as can be seen by the deteriorated sitting pig, the model may be of high relevance for the simulated mobilized ECMO patient. We believe that the selected model is valid for preclinical studies and that analog problems can be overcome for the human patient population. First and most importantly, in contrast to the animal model, awake patients are less sedated and should have a lesser degree of vasoplegia, i.e., a higher muscle tonus. The remaining vasoplegia can be further targeted by slow, moderated position changes of the patient and, analog to the abdominal compression of our model, with support stockings. We are positive that this concept is feasible as patients were already ambulated in several case studies. Third, we tried to blend ambient air with medical oxygen. Our data suggest that situations with a low oxygen demand may allow for resource saving gas blending, e.g., for more CO_2_-removal focused applications. Ideally, in combination with an automated control, the device measures oxygen and carbon dioxide levels in high frequency and adapts oxygen fraction and total gas flow continuously; an according system was published by our colleagues before [28]. With respect to our in vitro data, we could confirm that a high gas flow eliminates CO_2_ effectively. For ECCO_2_R-patients mainly suffering from hypercapnic respiratory failure, the portable gas blender may offer a large range extension.

In the experiments, severely hypoxic and hypercapnic animals were observed. The most limiting factor was the drainage insufficiency due to the small cannula size that prohibited higher ECMO flows. The optimal flow regime of the cannula is 1–1.5 L/min, i.e., half of the intended operating point. This caused low inlet pressures. Further, the NovaPort Twin cannula has only a single drainage opening either in superior or inferior vena cava. Both factors contribute to a limit in ECMO flow. Alternative cannulas, e.g., the Avalon cannula, are used for walking ECMO in a clinical setting and enable higher blood flow and consecutive oxygen transfer, but the application is limited due to high cannula migration and dislocation risk with severe complications [38,39,40,41,42,43,44]. We therefore conclude that the cannula was not well chosen for the intended use. In consequence, we were not able to show the functional limit of the membrane oxygenator unit. The current study design did not comprise the investigation of other cannulas. Our study showed the need for the investigation of the safe deployment of existing cannulas in different inlet and outlet positions in the relevant vessels, especially with respect to the altered hemodynamics of a sitting or mobilized patient. Due to the limitations of the currently available single- and dual-lumen cannulas, new concepts for the patient-ECMO interfaces seem necessary to provide efficient gas-transfer rates and simultaneously safe mobilization of the patient [45].

Blood gas analyses directly before and after the fiber bundle proved an effective gas transfer. Other contributive factors for the severe hypoxia and hypercapnia were, firstly, the low hemoglobin levels of around 10 g/dL. Secondly, the extracorporeal circulation may have been partly recirculating freshly oxygenated and decarboxylated blood; this phenomenon is common [46] and decreases the effective blood flow almost by the fraction of the recirculating blood [47,48]. Thirdly, the ratio of ECMO blood flow and native cardiac output usually ranges between approximately 1:5 and 1:12 from our experiences with similar animal models. Unfortunately, for the present study, we could not analyze the cardiac output due to a corrupted logging file of the conductance catheter monitor. Analyzing all available data from our experiment, we suggest that the extracorporeal oxygenation is mostly limited by the achievable extracorporeal blood flow. For the current proof of concept study with the chosen cannula, we aimed at a limited oxygen support and high carbon dioxide elimination. In theory, a lower support means less utilization of resources. The current study reflects the borderline of low flow within the applied animal model. While this was a plausible approach for the present study, it is necessary to run the system at an operating point prohibiting harmfully high carbon dioxide levels and a profound oxygen debt. A higher oxygen support of our device could be provided with alternative highflow cannulation strategies using available transatrial cannulas or other newly developed connection strategies.

The gas transfer rates ECMO VCO_2_ and ECMO VO_2_ showed very similar results to the in vitro tests for the respective blood flows. Müller et al. [49] as well as Kopp et al. [50] stated that 150 mL/min CO_2_-elimination is required to reach around 50% of the metabolic production for an ECCO_2_R scenario. We showed in our in vitro test higher values of around 160 mL/min; our in vivo test confirmed the results within the limited blood flow range due to the cannula.

### 4.3. Conclusions

Generally, the ambulation of ECMO patients may be beneficial, if existing obstacles can be resolved; these include: the reduction of procedural risks for nonimmobilized patients for the redefined therapy, integration of the system components, resource intensity (mainly staff), system autoregulation, the (prolongation of) temporary independence from static (hospital) infrastructure, proving safety and efficacy and therapeutic benefit of the concept, as for now, we rely on analogies from other therapies and single centers case studies and, last, the mitigation of the psychological burden of the patients.

Within the present study, we could show that the downsizing of an ECMO system with a carrying system close to the body, autonomous gas and energy supply, as well as a control system, is feasible. With the chosen cannula, the system was running rather like an ECCO_2_R device. A correctly selected cannula is obligatory to provide higher blood flow and, consecutively, higher oxygen transfer. Using a novel large animal model, we further showed the feasibility of a system applicable to mobilize patients and inherently established an appropriate in vivo model. We suggest progressing with the technology and the concept of mobilization to overcome the limitations of the laboratory sample. In order to conduct preclinical trials on safety and efficacy, even in animal studies, we must advance our system to reach at least industry standards. We therefore believe that a validation of our concept with improved system components and implemented industry standards is the definite next step.
